# Autoantibody to Interferon-gamma Associated with Adult-Onset Immunodeficiency in Non-HIV Individuals in Northern Thailand

**DOI:** 10.1371/journal.pone.0076371

**Published:** 2013-09-27

**Authors:** Panuwat Wongkulab, Jiraprapa Wipasa, Romanee Chaiwarith, Khuanchai Supparatpinyo

**Affiliations:** 1 Department of Medicine, Faculty of Medicine, Chiang Mai University, Chiang Mai, Thailand; 2 Research Institute for Health Sciences, Chiang Mai University, Chiang Mai, Thailand; Karolinska Institutet, Sweden

## Abstract

**Background:**

Autoantibody to interferon-gamma (IFN-γ) has been reported to be associated with adult-onset immunodeficiency in patients from Asian countries. This study aimed to determine the prevalence of autoantibody to IFN-γ among non-HIV patients in northern Thailand who were repeatedly infected with unusual intracellular pathogens.

**Methods:**

A cross-sectional, case-control study was conducted between March 2011 and March 2012 at Chiang Mai University Hospital. 20 cases, non-HIV, aged 18–60 years, presented with at least 2 episodes of culture or histopathology proven opportunistic infections were enrolled. Controls comprised 20 HIV-infected patients and 20 healthy adults who were age- and sex-matched with cases. Enzyme-linked immunosorbent assay (ELISA) was used to detect the presence of antibody to IFN-γ.

**Results:**

11 participants in each group were female. The mean ages were 48.1±6.4, 48.3±6.3, and 47.1±6.5 years among cases, HIV-infected, and healthy controls, respectively. The opportunistic infections among 20 cases included disseminated non-tuberculous mycobacterial (NTM) infection (19 patients/24 episodes), disseminated penicilliosis marneffei (12 patients/12 episodes), and non-typhoidal *Salmonella* bacteremia (7 patients/8 episodes). At the cutoff level of 99 percentile of controls, the prevalence of autoantibody to IFN-γ were 100%, 0%, and 0%, among cases, HIV-infected, and healthy controls, respectively (p-value <0.001). The mean concentrations of antibody to IFN-γ were 3.279±0.662 and 0.939±0.630 O.D. among cases with and without active opportunistic infection, respectively (p-value<0.001).

**Conclusions:**

In northern Thailand, autoantibody to IFN-γ was strongly associated with adult-onset immunodeficiency. The level of antibody to IFN-γ in patients who had active opportunistic infection was relatively higher than those without active infection.

## Introduction

Opportunistic infections caused by intracellular organisms, including non-tuberculous mycobacteria (NTM), *Cryptococcus neoformans*, *Penicillium marneffei,* and non-typhoidal *Salmonella* spp., have been observed in non-HIV individuals in many countries [1]–[12]. In the largest case series of these patients, from Thailand, the authors could not identify the cause of impaired cell-mediated immunity [3]. All were not HIV infected, had not received any immunosuppressive agents, and had no underlying diseases to explain compromise of their immune system. Recently, the cell-mediated immune deficiency in these HIV-negative, adult-onset immunodeficient patients was linked to the presence of autoantibody to interferon-gamma (IFN-γ) [1]–[2], [4]–[11]. IFN-γ is among the cytokines in the cell-mediated immune cascade produced in response to the invasion of intracellular infections [13]. It is mainly secreted by T-cells and natural killer (NK) cells to activate macrophages that can phagocytose and kill those intracellular pathogens [14]. The depletion of IFN-γ, perhaps by reduced secretion from T-cells and NK cells or by neutralization from antibody to IFN-γ, may impair the ability of intracellular organism killing by macrophages [15]. In HIV-infected individuals, the depletion of CD4+ T-cells is the major determinant for impaired cell-mediated immunity [16]. Although autoantibody to IFN-γ has been detected in HIV-infected individuals, it is present at a low level, and its clinical impact is still unclear [17].

**Figure 1:**
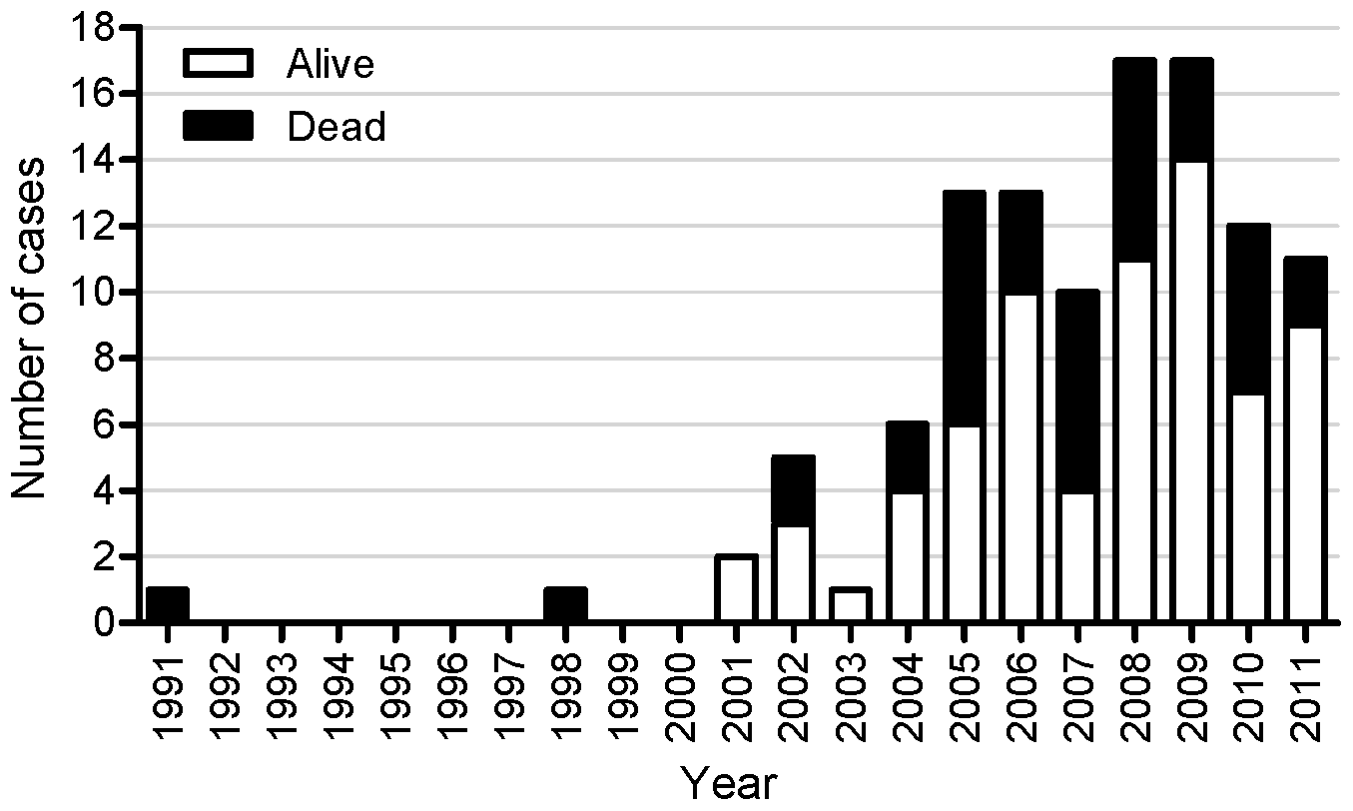
Patients with adult-onset immunodeficiency in northern Thailand (1991 to 2011).

In northern Thailand, an area with high prevalence of HIV infection, a number of non-HIV infected patients have recently presented with disseminated infections caused by unusual intracellular pathogens. From 1991 to 2011, 109 patients suspected of having adult-onset immunodeficiency were seen at Chiang Mai University Hospital. The first four cases were reported in 2007 [3]. Thereafter, 105 additional patients with similar clinical presentation were seen (Figure 1). We could not identify causes of cell-mediated immune deficiency, including HIV infection, in these patients. Although Browne et al has determined that the adult-onset immunodeficiency in patients from Thailand and Taiwan is strongly associated with high-titer neutralizing antibodies to IFN-γ [2], this phenomenon has never been studied in northern Thailand. Therefore, to confirm the hypothesis that the presence of autoantibody to IFN-γ is associated with cell-mediated immune deficiency in northern Thailand, we conducted a case-control study to compare the prevalence of antibody to IFN-γ among non-HIV infected individuals presenting with repeated episodes of disseminated infection caused by unusual intracellular pathogens, HIV-infected controls, and healthy controls. The secondary objectives were: 1) to describe the clinical characteristics of these patients with autoantibody to IFN-γ and 2) to compare the level of antibody to IFN-γ between patients with and without active opportunistic infection at study entry.

## Materials and Methods

### Study design and population

Over a 13-month period from March 2011 to March 2012, a prospective, cross-sectional, case-control study was conducted at Chiang Mai University Hospital, Chiang Mai, Thailand, an 1800-bed tertiary care facility serving all community and provincial hospitals throughout northern Thailand. The inclusion criteria for adult-onset immunodeficiency cases were 1) male or female between 18 to 60 years old; 2) negative for anti-HIV antibody; 3) presented with at least two episodes of culture or histopathology proven infections caused by unusual intracellular pathogens, i.e., NTM, disseminated fungal infections (e.g., penicilliosis marneffei, cryptococcosis, histoplasmosis), disseminated herpes zoster infection; and non-typhoidal *Salmonella* bacteremia; and 4) willing to participate the study by signing the informed consent. Cases with active opportunistic infection were defined as patients presenting with active infections during the past 30 days or those required intravenous antimicrobials to control the infections at study entry. Two groups of age- and sex-matched HIV-infected controls and healthy controls were enrolled for comparison. HIV infection was documented by enzyme-linked immunosorbent assay (ELISA) and confirmed by plasma HIV-1 RNA. Healthy controls were those without HIV infection and no history of 1) underlying medical conditions that may compromise immune status; 2) currently taking immunosuppressive agents; 3) mycobacterial infection; and 4) any active infections during the past 30 days. All cases and controls had to sign the informed consent before entry to the study.

### Data collection

Demographic data at enrollment including sex, age, residential area, and occupation were recorded. Clinical data including history of opportunistic infections, type of microbiologic diagnosis (staining, or culture), organ involvement, species of NTM (if available) were collected. Laboratory tests were uniformly performed in all patients, including complete blood count, blood urea nitrogen (BUN), serum creatinine, liver function test, anti-HIV antibody test, and CD4+ cell count. Patients’ sera were collected for assay of antibody to IFN-γ by methods described below.

### Screening for antibody to IFN-γ using ELISA

The screening for antibody to IFN-γ in serum was determined by enzyme-linked immunosorbent assay (ELISA) modified from the methods previously described by Tang et al. [11] One hundred microliters of 1 µg/ml recombinant human IFN-γ in bicarbonate buffer (pH 9.6) were added to a Maxisorb immunoplate (Nalge Nunc International). After overnight incubation at 4°C, plates were washed 4 times with washing buffer (0.05% Tween-20/PBS) and 200 µl of blocking buffer (1% skimmed milk/PBS) were added to each well. Plates were incubated at 37°C for 1 hour and supernatants were discarded. Patients’ sera were diluted 1000 times with blocking buffer, and 100 microliter was then added to each well for 1 hour incubation at 37°C. After washing for 4 times, 100 µl of 1∶2,000 goat anti-human IgG horseradish peroxidase conjugate (Caltag Laboratories, Invitrogen, Paisley, UK) were added to each well and plates were incubated at 37°C for 1 hour. After 4 washes, 100 µl of *o*-Phenylenediamine dihydrochloride substrate (Sigma) solution were added and plates were incubated at room temperature for 30 minutes. The enzyme reaction was terminated with sulphuric acid (2N) and absorbance was then read at 492 nm on a Spectra MR plate reader (Dynex Technology). The tests were repeated three times to confirm the laboratory results and the mean optical density (O.D.) of antibody to IFN-γ was used for analysis. Since there was no normal range as well as standard cut-off for the presence of antibody to interferon-gamma in Thai population, we used the 99th percentile of O.D.’s determined in sera from our HIV-infected and healthy controls as a cutoff for the presence of antibody to interferon-gamma; the O.D. of greater than this value was classified as positive for antibody to IFN-γ.

To determine the half maximal effective concentration (EC50), serum from each patient with positive antibody to IFN-γ was serially diluted four-fold from 1∶400 to 1∶ 6,553,600. The dilution of antibody that gave a response halfway between the baseline and maximum response of each individual was determined by derivation of curve-fitting software (GraphPad Prism). The mean EC50 among cases with and without active opportunistic infection were calculated and compared.

### Confirmation of antibody to IFN-γ using inhibition assay

Sera were incubated with different concentrations of recombinant human IFN-γ (R&D System) (ranging from 50 to 0.08 ng/ml) for 1 hour at 37°C. Unbound antibody to IFN-γ was then detected by ELISA as described above. This assay was performed only among cases to confirm the presence of antibody to IFN-γ in the patients’ sera.

### Statistical analysis

Descriptive data were presented in number (%), mean±SD, and median (IQR) as appropriate. Comparisons of clinical characteristics and laboratory data between case and each of controls were performed using Chi-square and Fisher’s exact test for categorical data and Student’s t-test or Mann-Whitney *U* test for continuous data as appropriate. All p-values were 2-sided at a significant level of 0.05. All analyses were performed using Stata statistical software version 10.0 (Stata Statistical Software: Release 10.0, Stata Corporation, College Station, TX, 2007).

#### Sample size calculation

Based on the assumption that the prevalence of IFN-γ autoantibody among cases was 80%, a minimum number of 14 subjects in each arm were estimated to detect the differences between 2 groups with the type I error of 0.05 and the power of 80% if the prevalence of IFN-γ autoantibody in each of the control groups was 30%. Although such high background prevalence in the normal population was unlikely, 20 cases were targeted for enrollment to ensure a more than adequate sample size to detect statistically significant difference. The study was approved by the Faculty of Medicine and Research Institute for Health Sciences, Chiang Mai University Ethical Committees.

## Results

### Study participants and demographic data

From March 2011 to March 2012, a total of 24 patients fulfilled the inclusion criteria of adult-onset immunodeficiency cases were enrolled. These patients resided in nine provinces around Northern Thailand. Four patients were excluded from the study; one patient was taking corticosteroid and three patients had only one episode of culture or histopathology proven infection. The other episodes were diagnosed only by microscopic examination. Most demographic characteristics were similar among three groups (Table 1), except the predominant residential area of Chiang Mai in the healthy controls and the predominant occupation of agriculture in cases. One patient in the cases group had previous history of Hodgkin’s disease but was in complete remission for 1 year without any immunosuppressive agent. Other underlying diseases among the cases group were Behcet’s disease and glucose-6-phosphate dehydrogenase (G6PD) deficiency (1 patient each). Among healthy controls, one patient each had polycystic kidneys, fibromyalgia, valvular heart disease, and obstructive sleep apnea.

**Table 1:** Demographic and clinical characteristics of the 60 participants.

Characteristics	Cases	HIV-infected controls	p-values†	Healthy controls	p-values‡
Age (yr)	48.1±6.4	48.3±6.3	1.000	47.1±6.5	0.63
Female sex−no. (%)	11 (55)	11 (55)	1.000	11 (55)	1.00
Body weight (kg)	52.3±11.6	56.5±11.4	0.118	59.9±9.4	0.027
Organ involvement*					
Lymph node	15				
Skin	14				
Bloodstream	11				
Lung	5				
Bone and joint	4				
Others	2				
Laboratory findings					
Hemoglobin (g/dL)	11.1±2.4	13.4±1.2	<0.001	13.5±1.9	<0.001
White blood cell count (×10^3^/µL)	12.6 (6.9, 19.2)	6.1 (4.7, 7.0)	<0.001	6.3 (5.2, 7.4)	<0.001
Absolute neutrophil count (×10^3^/µL)	7.7 (4.0, 12.5)	3.1 (2.5, 4.1)	<0.001	3.5 (2.8, 3.9)	<0.001
Absolute lymphocyte count (×10^3^/µL)	2.1 (1.7, 3.1)	1.9 (1.6, 2.2)	0.417	2.0 (1.8, 2.3)	0.72
Absolute eosinophil count (×10^3^/µL)	6.31 (4.15,10.31)	1.22 (0.85,3.41)	<0.001	1.78 (1.15,3.34)	<0.001
Platelet count (×10^3^/µL)	240.5 (199.5, 416.0)	244.0 (205.0, 282.0)	0.507	233.0 (197.0, 290.5)	0.30
CD4+ cell count (cells/mm^3^)	662 (418, 890)	435 (346, 550)	0.023	692 (624, 834)	0.42
Alkaline phosphatase (units/L)	103 (72, 228)	69 (63, 94)	0.015	57.5 (51.5, 67.0)	<0.001
Aspartate aminotransferase (units/L)	25 (21, 35)	32.5 (26, 39)	0.109	24 (20, 27)	0.25
Alanine aminotransferase (units/L)	20 (13, 28)	39 (24, 51.5)	0.004	22.5 (16.5, 27)	0.32

Data presented in number (%), means±SD, or median (IQR).

* Organ involvement show in each infectious episode, and one participant might have more than 1 site of infection.

† Compare between HIV-infected controls and cases.

‡ Compare between healthy controls and cases.

### Clinical characteristics and laboratory data

Table 1 shows the clinical characteristics of 20 adult-onset immunodeficiency cases. Most patients presented with multiple organ involvement, with lymph nodes as the most common organ involvement (15 patients, 75%). The second most common organ involvement was skin (14 patients, 70%). *Penicillium marneffei* was the most common causative organism involving skin (7 patients) followed by NTM (5 patients). The third most common clinical manifestation was bloodstream infection (11 patients, 55%); isolated organisms included non-tuberculous mycobacteria (6 patients), non-typhoidal *Salmonella* spp. (7 patients), and *Penicillium marneffei* (2 patients). Reactive skin lesions were present in 8 patients; 7 had neutrophilic dermatosis or Sweet's syndrome, one had generalized pustulosis and one had both.

For the laboratory findings, adult-onset immunodeficiency cases were more likely to have lower hemoglobin level and higher white blood cell count, absolute neutrophil count, absolute eosinophil count, and alkaline phosphatase than the control groups (Table 1). The absolute CD4+ cell count was comparable between cases and healthy controls but significantly higher than that in the HIV-infected controls.

Table 2 shows the prevalence of opportunistic infections among 20 cases and 20 HIV-infected controls. Among the 20 cases, disseminated NTM infection was the most common opportunistic infection (19 patients/24 episodes), followed by penicilliosis marneffei (12 patients/12 episodes), and non-typhoidal *Salmonella* bacteremia (7 patients/8 episodes). One patient each had cryptococcosis, histoplasmosis, and disseminated herpes zoster infection. Thirteen HIV-infected controls had previous history of opportunistic infections (15 episodes), most commonly *Pneumocystis* pneumonia (7 patients).

**Table 2:** Opportunistic infections among 20 cases and 20 HIV-infected controls.

Opportunistic infections*	Cases (N = 20)	HIV-infected controls (N = 20)
Infections caused by non-tuberculous mycobacteria	24	0
Rapid-grower mycobacteria	11	
*M. chelonae*	3	
*M. abscessus*	4	
*M. chelonae-abscessus complex*	3	
*M. fortuitum*	1	
Slow grower mycobacteria	7	
*M. avium*	3	
*M. avium/paratuberculosis/solitarium*	1	
*M. kansassii*	3	
Species not specified	6	
Penicilliosis marneffei	12	0
Cryptococcosis	1	0
Histoplasmosis	1	1
Non-typhoidal salmonellosis	8	0
Herpes zoster virus infection	1	2
Cerebral toxoplasmosis	0	1
*Pneumocystis jirovecii* pneumonia	0	7
Cytomegalovirus infection	0	2
Tuberculosis	0	2

* One participant might have more than 1 infectious episode.

### Prevalence of autoantibody to IFN-γ

**Figure 2:**
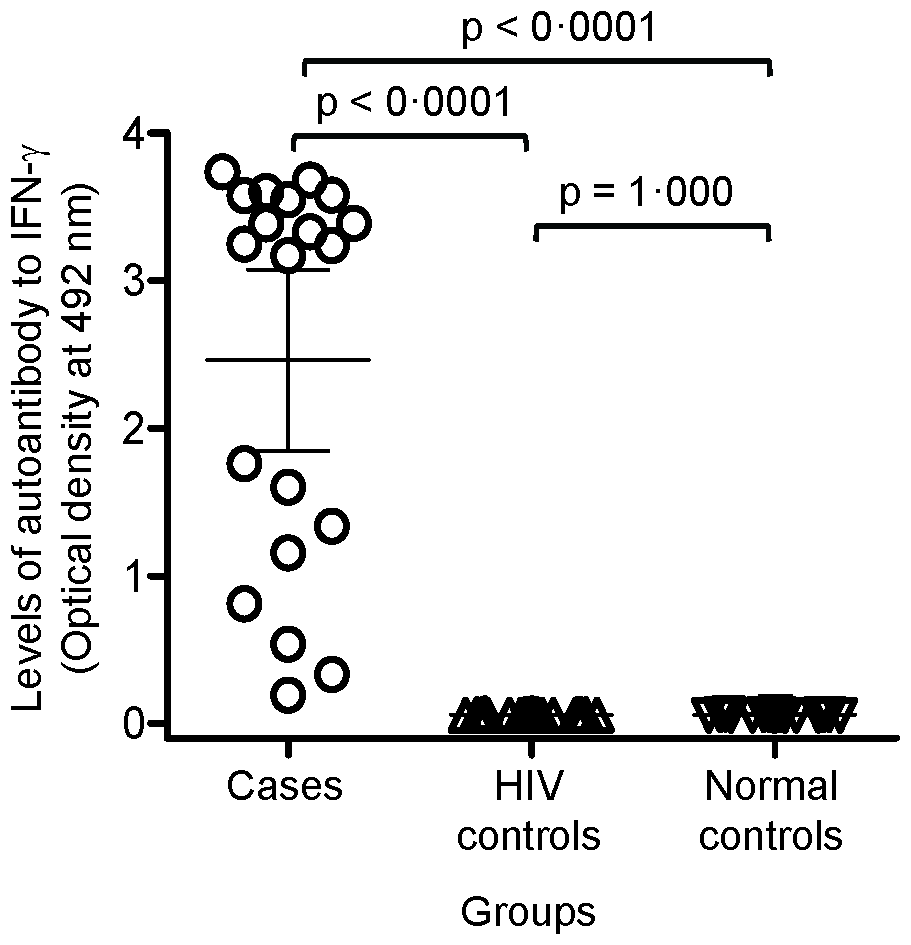
Levels of autoantibody to IFN-γ as determined by ELISA among 20 cases, 20 HIV-controls, and 20 healthy controls. Each symbol represents the data of one individual. Solid lines show the mean ± 95% confidence interval of each group.

Using the cutoff level at 99 percentile of the HIV-infected and the healthy controls, autoantibody to IFN-γ was present in 100%, 0%, and 0% among cases, HIV-infected patients, and healthy controls, respectively (p<0.001 between cases vs. each control group). Figure 2 shows the concentrations of autoantibody to IFN-γ among the three groups. The mean concentrations of autoantibody to IFN-γ were 2.460±1.309 O.D. among cases, 0.058±0.004 O.D. among HIV-infected controls (p<0.001), and 0.059±0.005 O.D. among healthy controls (p<0.001). The mean concentrations of autoantibody to IFN-γ were not different between HIV-infected controls and healthy controls (p = 1.000).

### Confirmation of antibody to IFN-γ using inhibition assay

**Figure 3:**
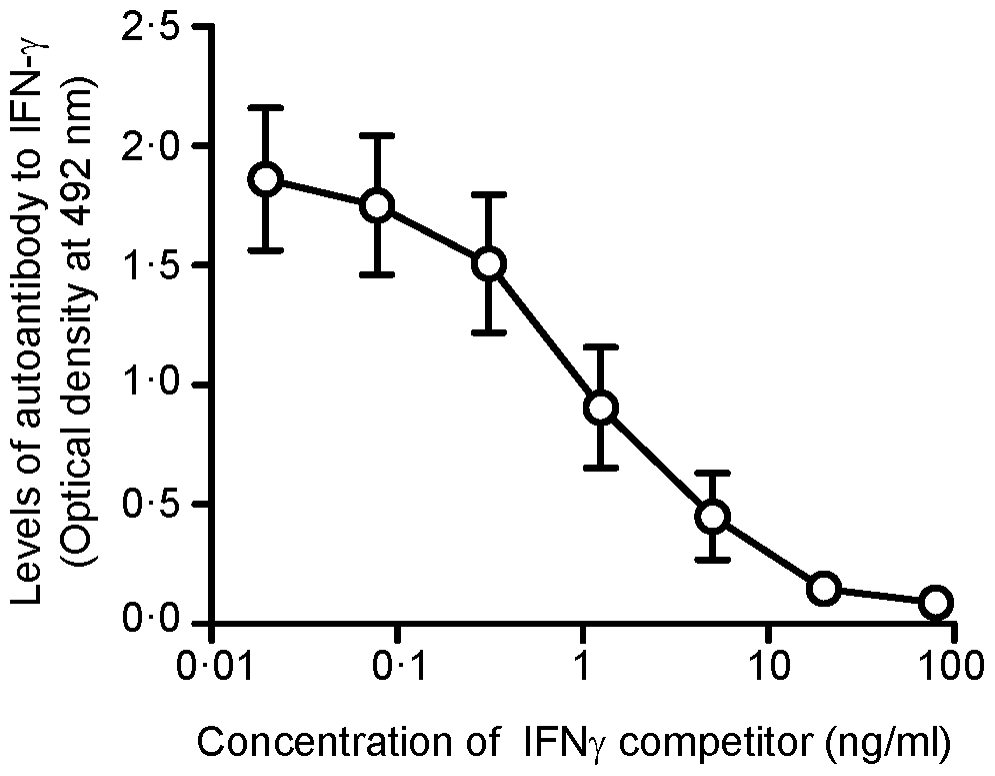
Inhibition assay for anti-IFN-γ antibody by competition with different concentrations of IFN-γ among 20 cases. Sera at 1∶2,000 dilution were incubated with different concentrations of recombinant human IFN-γ, ranging from 80 to 0.02 ng/ml for 1 hour at 37°C. Unbound anti-IFN-γ antibody was then detected by ELISA. The results show the average ± standard error of the means (SEM) O.D. values.

The inhibition assay was performed in all 20 cases; binding activity of autoantibody to IFN-γ was inhibited if sera were pre-incubated with soluble IFN-γ in a dose-dependent manner (Figure 3). This confirmed the presence of autoantibody to IFN-γ in all cases' sera.

### Clinical characteristics of cases with and without active opportunistic infection

Thirteen of 20 cases had active opportunistic infection at the study enrollment. The mean age of cases with active opportunistic infection was 47.0±6.7 years whereas the mean age of those without active opportunistic infection was 50.1±5.6 years (p = 0.306). The mean body weight of cases with active opportunistic infection was 48.7±11.0 and 59.0±10.1 kilograms among those without active opportunistic infection (p = 0.054). Seven of 13 (53.8%) and two of seven (28.6%) of cases with and without active opportunistic infection were male (p = 0.279). The mean concentrations of autoantibody to IFN-γ was significantly higher among those with active opportunistic infection (3.279±0.662 O.D.) compared with those without active opportunistic infection (0.939±0.630 O.D., p<0.001). The mean EC50 in those with and without active opportunistic infection were 10883±13024 and 1266±53.22, respectively (p<0.001). The white blood cell count, absolute neutrophil count, absolute CD4+ cell count, and level of alkaline phosphatase were significantly higher among those with active opportunistic infection compared with those without active opportunistic infection. (Table 3).

**Table 3:** Laboratory data in cases with and without active opportunistic infection.

Laboratory	Cases with active opportunistic infection (N = 13)	Cases without active opportunistic infection (N = 7)	p-values
Hemoglobin (g/dL)	10.2±2.2	12.7±1.8	0.022
White blood cell count (×10^3^/µL)	15.3 (13.3,21.1)	6.7 (5.0,7.0)	0.001
Absolute neutrophil count (×10^3^/µL)	11.7 (9.5,15.1)	3.4 (3.0,4.6)	0.001
Absolute lymphocyte count (×10^3^/µL)	2.3 (1.7,3.3)	2.0 (1.7,2.2)	0.25
Absolute eosinophil count (×10^3^/µL)	8.3 (5.7, 11.7)	3.0 (1.3, 5.2)	0.008
Platelet count (×10^3^/µL)	340.0 (247.0,498.0)	218.0 (193.0,232.0)	0.052
Absolute CD4 count (cells/mm^3^)	750 (460,925)	432 (306,654)	0.088
Erythrocyte sedimentation rate (mm/hr)	87.8±44.8	48.5±51.9	0.11
Alkaline phosphatase (units/L)	164(103,235)	75.5(55,90)	0.010
Aspartate aminotransferase (units/L)	25 (21,35)	28.5 (21,35)	0.79
Alanine aminotransferase (units/L)	20 (15,29)	15.5 (13,23)	0.45

Data presented in means±SD or median (IQR).

## Discussion

Our study, the first report from northern Thailand, confirms that the number of non-HIV infected patients with adult-onset immunodeficiency are recently increasing in Asian population. Retrospective data collection from 109 patients who were suspected of having adult-onset immunodeficiency at Chiang Mai University Hospital between 1991 and 2011 showed that this newly recognized syndrome was slightly more common in female with the mean age at the time of first diagnosis of 49 years. These patients had a relatively high mortality, i.e., 32% died at the median time of 25 months after diagnosis. There has been no longitudinal study to compare survival rate; however, Tang et al also observed a poor prognosis in patients with adult-onset immunodeficiency [11].

This study found further evidence that autoantibody to IFN-γ may play an important role in cell-mediated immunity defect among non-HIV infected Thai patients in northern Thailand with repeated episodes of unusual intracellular infections, as reported elsewhere [1]–[2], [4]–[6], [8]–[11]. Using the cutoff O.D. at 99 percentile of HIV-infected and healthy controls, all cases in this study were positive for autoantibody to IFN-γ, whereas neither HIV-infected controls, many of which had history of similar opportunistic infections, nor healthy controls had IFN-γ autoantibody. These findings were similar to those reported by Browne et al, who found high-titer anti–IFN-γ autoantibodies in 88% of patients with disseminated NTM infection or other opportunistic infections, compared with 2% of patients with tuberculosis and healthy controls [2]. Our study differs from Browne’s. First, our participants reside in northern Thailand, whereas Browne et al enrolled those from central and northeastern Thailand and from Taiwan. Second, we included only patients with at least 2 episodes of culture or histopathology proven infections caused by unusual intracellular pathogens, to more accurately confirm the true immunocompromised state. This could exclude the possibility of enrollment of subjects with normal or slightly impaired immune function. As the result, our study demonstrated a 100% prevalence of high-titer autoantibody to IFN-γ compared with 88% in the study by Browne et al. The second group in Browne’s study that included patients with opportunistic infection with or without NTM was similar to our cases, resulting in a 98% prevalence. Third, our study had smaller number of cases with adult-onset immunodeficiency, i.e., 20 vs. 97 cases in the study by Browne et al. However, as stated in the Sample Size Calculation section, only 14 subjects in each arm were required to detect the statistical difference despite the overestimate of very high background prevalence of IFN-γ autoantibody in the control groups. The enrollment of 20 cases is adequate to detect a statistically significant difference. Fourth, our study was designed to enroll controls who were age- and sex- matched with the cases group. This can minimize the selection bias since age and sex may influence the integrity of immune status. Fifth, we also included the HIV-infected controls for comparison. Although there were reports of the presence of low-level autoantibody to IFN-γ among HIV-infected individuals, impaired cell-mediated immune response in these patients was mainly from CD4+ cell depletion leading to the reduction of cytokines including IFN-γ [17]. The 0% prevalence of autoantibody to IFN-γ in HIV-infected control in our study confirms this hypothesis. Sixth, all the healthy controls in our study were tested and confirmed to be negative for HIV infection while not uniformly done by Browne et al. Finally, we used a relatively simple method of ELISA for detection and quantification of autoantibody to IFN-γ while Browne et al used methods that need an advanced laboratory and skillful technicians. Our method is easily developed in general laboratory especially in the resource limited settings. The commercial kit could be developed and marketed at a low cost in the near future. We also plan to develop a simple point-of-care test kit to detect the presence of autoantibody to IFN-γ in the serum based on this assay.

Our study found that NTM was the most common cause of opportunistic infections in patients with adult-onset cell-mediated immune deficiency; this is similar to the previous case reports and case series of similar patients [1]–[2], [4]–[11]. However, the second most common opportunistic infection in our study is disseminated penicilliosis marneffei. This systemic fungal infection is endemic in Northern Thailand and is among the most common opportunistic infections in HIV-infected individuals in this region [18]. Another report from Taiwan also showed the high prevalence of disseminated penicilliosis marneffei [11]. Physicians in the region should aware of this adult-onset cell-mediated immune deficiency associated with high-titer neutralizing antibodies to IFN-γ in non-HIV individuals who presented with disseminated penicilliosis marneffei.

We also did a subgroup analysis among cases to compare the clinical characteristics and laboratory findings between those with and without active opportunistic infection at study entry. Despite a small number of participants, we found several laboratory parameters correlated with the disease activity including the higher white blood cell count and absolute neutrophil count, as well as lower hemoglobin level. Interestingly, the level of autoantibody to IFN-γ in patients with active infections was relatively higher than those without active infections. Therefore, in addition to be a tool for diagnosis, the level of autoantibody to IFN-γ may have a role in monitoring the disease activity or recurrence of the disease. This will be helpful for the evaluation of treatment response in the future intervention trials in patients with adult-onset cell-mediated immune deficiency.

Our study has some limitations. First, we did not perform a complete immunological evaluation including laboratory tests for all cytokines responsible to intracellular infections. However, the study by Browne et al [2], which performed the tests for 41 anticytokine autoantibodies, demonstrated that only the autoantibody to IFN-γ distinguished patients with opportunistic infections from the other groups. Second, the sample size of our study was quite small. However, with the prevalence of autoantibody to IFN-γ among the control groups of 0% and the prevalence among cases of 100% and type I error of 0.05, we would have a power of 100% to detect the difference between 2 groups if it is existed. Third, our cross-sectional study analyzed the data from single serum specimen collected from each patient. Longitudinal data collection is essential to determine if the autoantibody to IFN-γ remains high when patients are cured from infections. Fourth, our study used an ELISA method to detect the antibodies against INF-γ. The antibody concentrations derived by this method (in O.D. unit or titer) may not accurately quantify the levels of antibodies. Further study on quantitative assay is needed, particularly when the method is used in the longitudinal study or as a monitoring tool for future therapeutic intervention trials.

In conclusion, we confirm the strong association between the adult-onset immunodeficiency and the presence of autoantibody to IFN-γ in non-HIV individuals in northern Thailand. The level of antibody to IFN-γ in patients who had active opportunistic infection was relatively higher than those without active infection. Although the biological function of the autoantibody to IFN-γ has not been clearly identified, patients with this syndrome continue to suffer from opportunistic infections despite antimicrobial therapy. Therapeutic interventions targeting antibody production by B-cells and/or neutralization of autoantibody to IFN-γ may warrant investigation.
